# Glycosylation engineering of therapeutic IgG antibodies: challenges for the safety, functionality and efficacy

**DOI:** 10.1007/s13238-017-0433-3

**Published:** 2017-06-08

**Authors:** Yusuke Mimura, Toshihiko Katoh, Radka Saldova, Roisin O’Flaherty, Tomonori Izumi, Yuka Mimura-Kimura, Toshiaki Utsunomiya, Yoichi Mizukami, Kenji Yamamoto, Tsuneo Matsumoto, Pauline M. Rudd

**Affiliations:** 1 Department of Clinical Research, NHO Yamaguchi-Ube Medical Center, 685 Higashi-Kiwa, Ube, 755-0241 Japan; 2 Laboratory of Molecular Biology and Bioresponse, Division of Integrated Life Science, Graduate School of Biostudies, Kyoto University, Kitashirakawa, Oiwake-Cho, Sakyo-Ku, Kyoto 606-8502 Japan; 3 NIBRT GlycoScience Group, National Institute for Bioprocessing Research and Training, Mount Merrion, Blackrock, Dublin 4, Ireland; 4 Center for Regenerative Medicine, Yamaguchi University Graduate School of Medicine, 1-1-1 Minami Kogushi, Ube, 755-8505 Japan; 5 Center for Gene Research, Yamaguchi University, 1-1-1 Minami-Kogushi, Ube, 755-8505 Japan; 6 Research Institute for Bioresources and Biotechnology, Ishikawa Prefectural University, 1-308 Suematsu, Nonoichi, Ishikawa 921-8836 Japan

**Keywords:** chemoenzymatic glycoengineering, crystal structure, endoglycosidase, fucose, glycosylation, intravenous immunoglobulin, sialic acid, transglycosylation, ultra performance liquid chromatography

## Abstract

Glycosylation of the Fc region of IgG has a profound impact on the safety and clinical efficacy of therapeutic antibodies. While the biantennary complex-type oligosaccharide attached to Asn297 of the Fc is essential for antibody effector functions, fucose and outer-arm sugars attached to the core heptasaccharide that generate structural heterogeneity (glycoforms) exhibit unique biological activities. Hence, efficient and quantitative glycan analysis techniques have been increasingly important for the development and quality control of therapeutic antibodies, and glycan profiles of the Fc are recognized as critical quality attributes. In the past decade our understanding of the influence of glycosylation on the structure/function of IgG-Fc has grown rapidly through X-ray crystallographic and nuclear magnetic resonance studies, which provides possibilities for the design of novel antibody therapeutics. Furthermore, the chemoenzymatic glycoengineering approach using endoglycosidase-based glycosynthases may facilitate the development of homogeneous IgG glycoforms with desirable functionality as next-generation therapeutic antibodies. Thus, the Fc glycans are fertile ground for the improvement of the safety, functionality, and efficacy of therapeutic IgG antibodies in the era of precision medicine.

## Introduction

Glycosylation of proteins is a complex and versatile posttranslational modification that influences biological activity, protein conformation, stability, solubility, secretion, pharmacokinetics, and antigenicity (Dwek, 1998). IgG is composed of three globular domain structures, two of which are the fragments for antigen binding (Fab) and the other is the fragment crystalizable (Fc) that activates Fcγ receptors (FcγRs) on leukocytes and C1 component of complement. IgG molecules bear oligosaccharides at Asn297 of the Fc region, and the oligosaccharide plays an essential role in Fc effector functions including antibody-dependent cellular cytotoxicity (ADCC) and complement-dependent cytotoxicity (CDC) that are among mechanisms of action of therapeutic antibodies. Therefore, engineering of Fc glycosylation is a rational strategy to improve the safety and efficacy of therapeutic IgG antibodies. Although the importance of glycosylation for Fc effector functions of therapeutic IgG antibodies has been previously documented (Jefferis, 2009, 2012, 2017; Mimura et al., 2009; Zhang et al., 2016), this review summarizes recent advances in antibody glycobiology that are applicable for optimization of the functionality of IgG antibodies for therapeutic purposes, including the novel glycan profiling technology developed by Rudd’s group (Bones et al., 2010), the influence of glycosylation on the structure and function of the Fc revealed from crystal structures of nonglycosylated Fc, the nonfucosylated Fc-glycosylated Fcγ receptor IIIa (FcγRIIIa) complexes and sialylated Fc and a new approach to engineering of IgG glycoforms via transglycosylation of predefined oligosaccharides to deglycosylated IgG-Fc.

## Structure of the IgG-Fc glycans

**Figure 1:**
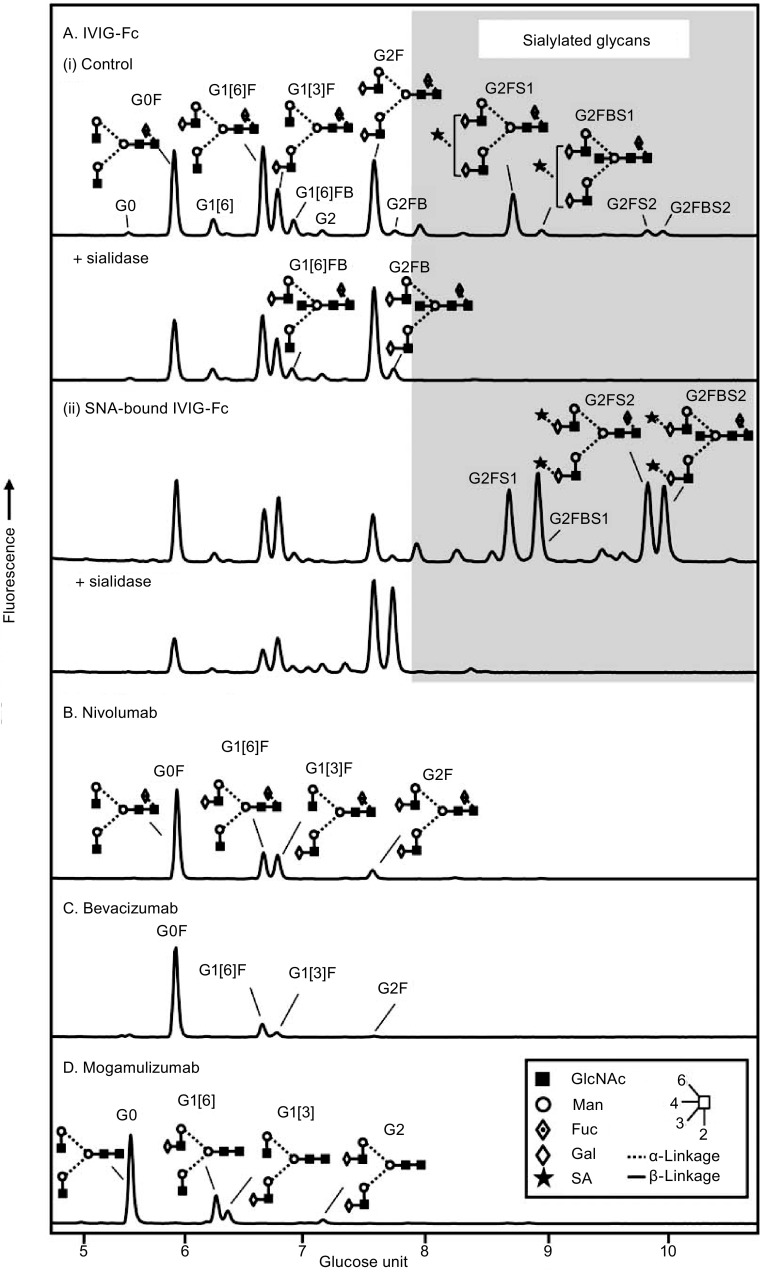
**Glycan profiles of therapeutic antibodies by hydrophilic interaction liquid chromatography (HILIC)**. The glycan profiles of the Fc of IVIG (A). The control IVIG-Fc (i) and the SNA-bound IVIG-Fc fraction (ii). The glycan profiles of therapeutic IgG monoclonal antibodies (B–D). (B) Nivolumab (human anti-PD-1 IgG4), (C) Bevacizumab (humanized anti-VEGF IgG1), (D) Mogamulizumab (humanized anti-CCR4 IgG1). The glycans were released with peptide-N-glycosidase F from the Fc fragments of IVIG and the heavy chains of the recombinant IgG antibodies in the SDS-PAGE gel bands and labeled with 2-aminobenzamide as previously described (Royle et al., 2006). The fluorescently labeled glycans were separated by ultra-performance liquid chromatography (UPLC) on a sub-2 μm hydrophilic interaction based stationary phase with a Waters Ethylene Bridged Hybrid (BEH) Glycan chromatography column (150 × 2.1 mm i.d., 1.7 μm BEH particles) (Bones et al., 2010; Doherty et al., 2012). The glycan peaks were assigned in accordance with the previous study (Pucic et al., 2011). Glycans are designated by the letters G, F, S, and B indicating the presence of galactose, fucose, sialic acid, and bisecting GlcNAc, respectively. [3] and [6] in the G1 glycan codes indicate the attachment of galactose on the α(1-3)- and α(1-6)-arm, respectively. Symbols of monosaccharides and lines for showing glycosidic linkages (Inset)

The IgG-Fc glycans released from human normal polyclonal IgG are highly heterogeneous (Arnold et al., 2006; Mimura et al., 2009; Rudd and Dwek, 1997) (Fig. 1A), due to the variable addition of fucose, bisecting GlcNAc, galactose, and sialic acid residues to the core complex biantennary heptasaccharide (GlcNAc_2_Man_3_GlcNAc_2_, designated G0). The heterogeneous glycans can be classified into three sets (G0, G1, and G2), depending on the number of galactose residues in the outer arms of biantennary glycans. Within each of these sets are four species that result from the presence or absence of core fucose and bisecting GlcNAc, namely, 16 neutral complex-type structures. Figure 1A shows the glycan profile of intravenous immunoglobulin (IVIG, Kenketsu Venilon-I, Teijin Phama) which is a therapeutic preparation of polyclonal IgG derived from pooled plasma of thousands of healthy donors. The fluorescently labeled glycans from the Fc fragment of IVIG (IVIG-Fc) were separated into >20 peaks by hydrophilic interaction liquid chromatography (HILIC) in which fucosylated, monogalactosylated (G1F) glycoforms predominate, with a preference for galactosylation on the α(1-6)-arm (G1[6]F) over the α(1-3)-arm (Fig. 1A-i, Table 1) (Pucic et al., 2011). The proportion of the sialylated glycoforms of IVIG-Fc was approximately 19% (Table 1), and sialic acid is known to be added preferentially on the α(1-3)-arm of the digalactosylated (G2) glycoforms (Barb et al., 2009; Grey et al., 1982; van den Eijnden et al., 1980). Sialylation occurs in α(2-6)-linkage with N-acetylneuraminic acid (NeuAc) in humans whereas it is in α(2-3)-linkage in Chinese hamster ovary (CHO)-derived recombinant IgG molecules (Takeuchi et al., 1988). The glycan profile of *Sambucus nigra* agglutinin (SNA)-bound IVIG-Fc showed the prominent peaks of monosialylated and disialylated glycans with and without bisecting GlcNAc (>60%, Fig. 1A-ii, Table 1). The presence of around 40% of non-sialylated glycans released from the SNA-bound Fc indicates that sialylation of one of the two Fc glycans is sufficient for the Fc to bind to SNA. The glycans of the therapeutic monoclonal IgG antibodies nivolumab (Opdivo^®^, Ono Pharmaceutical), bevacizumab (Avastin^®^, Chugai Pharmaceutical), and mogamulizumab (Poteligeo^®^, Kyowa Hakko Kirin) were less heterogeneous than those of IVIG-Fc (Fig. 1B–D). Currently approved therapeutic IgG antibodies are produced in CHO, NS0 and Sp2/0 cell lines, and nivolumab and bevacizumab are produced in CHO cells (Fig. 1B and C) and mogamulizumab in α(1-6)-fucosyltransferase (FUT8)-deficient CHO cells (Fig. 1D), which clearly shows the presence and absence of core fucose, respectively. Non-galactosylated glycoforms (G0F and G0) predominated, and sialylated glycans were negligible for these CHO-derived IgG antibodies (Table 1). Recombinant IgG antibodies produced from CHO and murine cells do not contain bisecting GlcNAc in contrast to human IgG as observed for IVIG (Fig. 1A) (Raju et al., 2000).

**Table 1:** Analysis of the key features of the *N*-glycans released from the therapeutic IgG antibodies^a^

IgG	Sialylation (%)		Term. Gal (%)^b^	Term. GlcNAc (%)^c^	Bisecting GlcNAc (%)	Core fucose (%)	Predominant glycoform
	S1	S2					
IVIG-Fc	16.2	2.6	62	19.2	11.4	92.8	G1F
SNA-IVIG-Fc	32.5	30.2	27.2	10.1	36.1	89.4	G2FBS1
Nivolumab	1.3	0	42.4	56.3	0	100	G0F
Bevacizumab	0	0	19	81	0	97.4	G0F
Mogamulizumab	0	0	36.3	63.7	0	0	G0

^a^Glycans were quantitated by measuring peak areas in the HILIC profiles (Fig. 1)

^b^Glycoforms terminating in galactose residues (G1, G1F, G1FB, G2, G2F, G2FB)

^c^Glycoforms terminating in GlcNAc residues (G0 and G0F)

Terminal α(1-3)-linked galactose (α(1-3)-Gal) and N-glycolylneuraminic acid (NeuGc) residues are frequently found in the *N*-glycans of recombinant IgG antibodies produced from murine myeloma cells. Such glycan structures are unnatural and potentially immunogenic in humans. The α-galactosylation and sialylation with NeuGc are reported in cetuximab produced from Sp2/0 (Qian et al., 2007) and infliximab from NS0 (Mimura et al., 2009) and are markedly increased for an IgG1-F243A mutant when expressed in murine cells (Mimura et al., 2016). It has been reported that all humans have IgG antibodies specific for the α(1-3)-Gal epitope (Galili et al., 1993) and that the anti-NeuGc activity is detectable in up to 85% of healthy individuals (Tangvoranuntakul et al., 2003; Zhu and Hurst, 2002). Cetuximab bears glycans containing both α(1-3)-Gal (30%) and NeuGc (12%) on the Fab portion (Qian et al., 2007), and there is a high prevalence of anti-α(1-3)-Gal IgE antibody in areas of the United States where anaphylactic reactions to cetuximab have occurred (Chung et al., 2008). Attempts have been made to predict severe cetuximab-induced hypersensitivity reactions prior to exposure to cetuximab (Iwamoto et al., 2016; Mariotte et al., 2011).

## Influence of the Fc glycan on antibody effector functions

**Figure 2:**
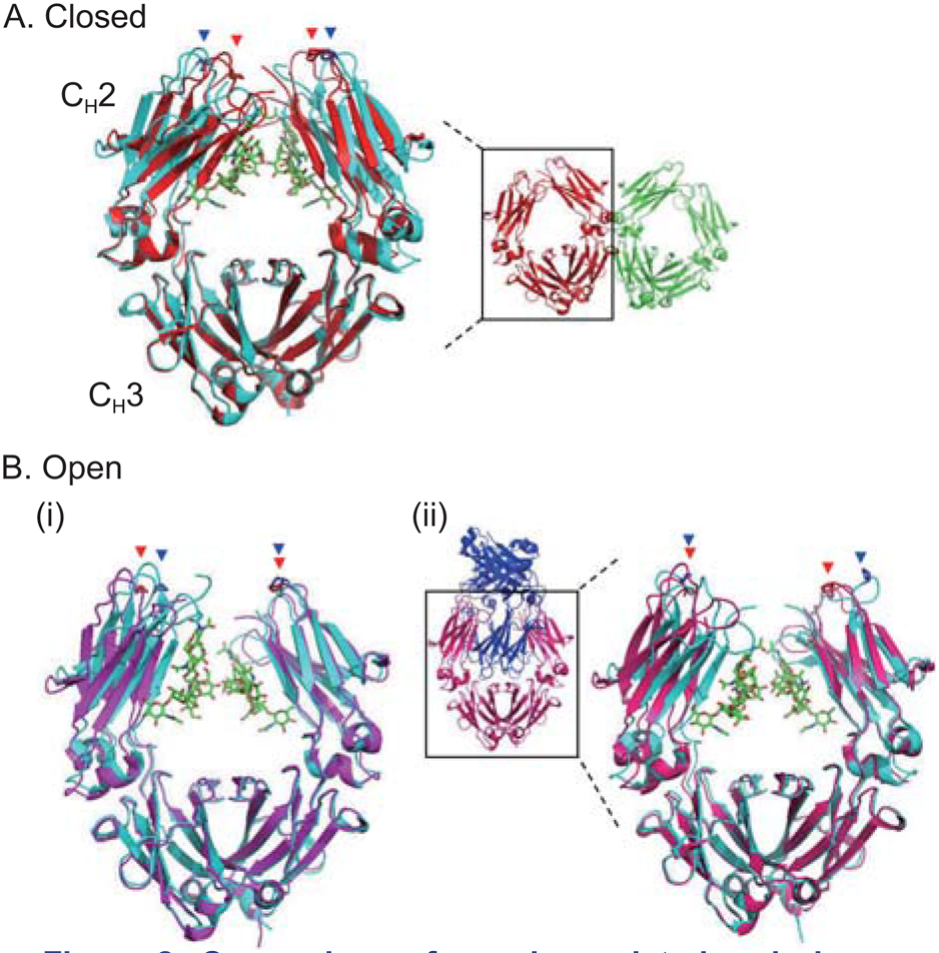
**Comparison of nonglycosylated and glycosylated Fc structures**. (A) Closed conformation of the nonglycosylated Fc. Superposition of the *E*. *coli*-produced aglycosylated human IgG1-Fc (red) (PDB ID code: 3S7G) with the glycosylated human IgG1-Fc (cyan) (PDB ID code: 3AVE). Overall structure of the two aglycosylated Fc molecules is shown in red and green, and the Fc shown in red is superimposed with the glycosylated Fc. (B) Open conformation of the nonglycosylated Fcs. (i) Superposition of the enzymatically deglycosylated human IgG1-Fc (magenta) (PDB ID code: 3DNK) with the glycosylated human IgG1-Fc (cyan) (PDB ID code: 3AVE). (ii) Superposition of deglycosylated human IgG4-Fc myeloma protein Rea (pink) (PDB ID code: 4D2N) with the glycosylated IgG4-Fc (cyan) (PDB ID code: 4C54). Overall structure of the two interlocked Fc molecules is shown in pink and blue. The Fc shown in pink is superimposed with the glycosylated Fc. The Fc glycans are shown in green sticks. The Pro329 residues located in the FG loop of the C_H_2 domains are indicated by red and blue arrowheads for the nonglycosylated and glycosylated C_H_2 domains, respectively. The molecular models were produced with PyMOL (The PyMOL Molecular Graphics System, Version 1.8.5.0, Schrodinger, LLC)

The oligosaccharides at Asn297 of the IgG-Fc are essential for the optimal activation of FcγRs and complement C1 although the carbohydrate moiety accounts for only 2%–3% of the IgG molecule. The clearance mechanisms including phagocytosis, ADCC, and CDC mediated by Fcγ receptors and C1q are abrogated or severely compromised for aglycosylated or deglycosylated forms of IgG (Nose and Wigzell, 1983; Pound et al., 1993; Sarmay et al., 1992; Tao and Morrison, 1989; Woof and Burton, 2004). The IgG-Fc crystal structure reveals the oligosaccharide as integral to the Fc structure, sequestered in the internal space enclosed by the two C_H_2 domains (Fig. 2. Glycans shown as green sticks). The electron density map provides coherent diffraction for the monogalactosylated oligosaccharide and allows the possibility of >70 contacts with 14 amino acid residues of the C_H_2 domain (Deisenhofer, 1981; Padlan, 1990). The crystal structure of the complex between IgG1-Fc and an *E*. *coli*-produced soluble recombinant form of FcγRIII (sFcγRIII) has demonstrated that the FcγRIII binds to the lower hinge and the hinge proximal regions of the two C_H_2 domains asymmetrically with a 1:1 stoichiometry (Radaev et al., 2001; Sondermann et al., 2000). In the complex of the Fc with the aglycosylated sFcγRIII, the Fc glycans are not directly associated with sFcγRIII except the primary GlcNAc of one oligosaccharide although removal of the Fc glycans abrogates sFcγRIII binding. Importantly, the interaction between IgG-Fc and sFcγRIII can be substantially influenced by the presence or absence of fucosylation of the Fc and glycosylation of sFcγRIII (see below) (Ferrara et al., 2011; Mizushima et al., 2011). Notably, the horseshoe-shaped Fc opens up upon complex formation, and therefore it is presumed that the Fc glycan maintains the open conformation of the Fc and that removal of the Fc glycan results in a closed conformation. This notion is supported by the crystal structures of the Fc glycoforms bearing sequentially truncated glycans ((G2F)_2_, (G0F)_2_, (M3N2F)_2_ and (MN2F)_2_, G: galactose; M: mannose; N: GlcNAc; F: fucose) in which the (G2F)_2_ glycoform shows the longest Pro329-Pro329 Cα distance of 33.7 Å whereas the (MN2F)_2_ glycoform the shortest distance of 21.9 Å (Krapp et al., 2003). Although truncation of the terminal sugar residues results in an increase of destabilization of the C_H_2 domain and a reduction of affinity to sFcγRIIb (Mimura et al., 2001) and sFcγRIII (Yamaguchi et al., 2006), the profound influence of Fc glycosylation on FcγR binding has not been paralleled by gross conformational differences between glycosylated and aglycosylated Fc fragments (Lund et al., 1990).

## Structures of nonglycosylated IgG-Fc

Recently crystal structures have been solved for *E*. *coli*-expressed, aglycosylated murine IgG1-Fc (PDB ID code: 3HKF) and aglycosylated human IgG1-Fc (PDB ID code: 3S7G) (Fig. 2A) (Borrok et al., 2012) and enzymatically deglycosylated human IgG1-Fc (PDB ID code: 3DNK) (Fig. 2B-i) and deglycosylated IgG4-Fc (PDB ID code: 4D2N) (Fig. 2B-ii) (Davies et al., 2014a). The bacterially expressed murine aglycosylated IgG1-Fc shows a strongly closed conformation (Feige et al., 2009). The crystal structure of aglycosylated human IgG1-Fc (PDB ID code: 3S7G) reveals two Fc dimers of the asymmetric unit, interfacing at the C_H_2-C_H_3 elbow between the dimers, and adopts a closed Fc conformation with Pro329-Pro329 Cα distances of 18.9 Å and 19.6 Å for the two Fc molecules whereas the structure of native Fc (PDB ID code: 3AVE) shows the Pro329-Pro329 distance of 25.1 Å (Fig. 2A) (Borrok et al., 2012). Furthermore, significant disorder is observed in the C’E loop (Gln293–Phe303) that contains the *N*-glycosylation site and a region crucial for FcγR binding. In contrast, the crystal structure of enzymatically deglycosylated human IgG1-Fc (PDB ID code: 3DNK) reveals an open conformation (Pro329–Pro329 distance, 27.6 Å) (Fig. 2B-i). The crystal structure of the enzymatically deglycosylated human IgG4-Fc myeloma protein (Rea) reveals two interlocked Fc molecules with the C_H_2 domains oriented in a symmetric open conformation (Pro329–Pro329 distance, 29.1 Å) (Fig. 2B-ii) (Davies et al., 2014a). There are no significant differences between the overall structures of deglycosylated IgG4-Fc and glycosylated IgG4-Fc although the conformation of the C’E loop is altered in the absence of the oligosaccharide (Davies et al., 2014a; Davies et al., 2014b). Thus, it seems likely that nonglycosylated C_H_2 domains can adopt not only closed but also flexible orientations. Furthermore, the aglycosylated human IgG1-Fc in Fig. 2A displays larger radii of gyration than glycosylated Fc by small angle X-ray scattering, which suggests a more open C_H_2 domain conformation in solution (Borrok et al., 2012).

## Aglycosylated IgG antibodies for therapy

Aglycosylated antibodies are suited for purposes where ADCC/CDC action is not required as is the case for neutralizing, agonistic or antagonistic antibodies. Numerous aglycosylated IgG antibodies are under clinical evaluation including otelixizumab (TRX4), onartuzumab (MetMAb), and clazakizumab (ALD518) (Ju and Jung, 2014; Jung et al., 2011). The use of aglycosylated IgG antibodies provides the following advantages: (1) The serum half-life of aglycosylated IgG is shown to be comparable to that of glycosylated counterpart in chimpanzees (Simmons et al., 2002); (2) Aglycosylated IgG can be produced in lower eukaryotes or in bacteria, which provides bioprocessing advantages in terms of shorter bioprocess development and running times without need to consider glycan heterogeneity problems; (3) Aglycosylated IgG antibodies maintain the ability to engage some of the FcγRs by a small subset of substitutions in the C_H_2 and/or C_H_3 domains. An aglycosylated IgG variant with S298G/T299A mutations has been identified that activates FcγRIIa (Sazinsky et al., 2008). In addition, an aglycosylated IgG variant with mutations E382V/M428I within the C_H_3 domain has been shown to mediate cytotoxicity of target cells via FcγRI (Ju et al., 2015; Jung et al., 2010). Thus, engineering of aglycosylated IgG provides new routes for the design of therapeutic antibodies with customized functionality.

Deglycosylation of circulating IgG* in vivo* by administration of endoglycosidase from *Streptococcus pyogenes* (Endo-S) has been considered as a novel therapeutic strategy for immune evasion in patient with autoimmune disorders (Allhorn and Collin, 2009; Collin et al., 2008). Endo-S selectively hydrolyzes the glycosidic bond of the chitobiose core of the Fc glycans leaving the primary GlcNAc with or without fucose, and Endo-S treatment of IgG results in a severely reduced affinity to FcγRs. Administration of recombinant Endo-S to mice has been shown to transiently remove the Fc glycans from circulating IgG and suppress inflammation in autoimmune models including immune thrombocytopenic purpura (ITP) and serum transfer arthritis (Albert et al., 2008). As a novel approach to enhance the efficacy of therapeutic antibodies, both Endo-S and therapeutic IgGs bearing Endo-S-resistant high mannose-type glycans are administered to eliminate competition for FcγR binding between circulating IgG and therapeutic IgG so that the therapeutic IgG could efficiently exert effector functions (Baruah et al., 2012). However, repeated administrations of the bacteria-derived endoglycosidase may lead to the development of antibodies against the enzyme. It should also be noted that immune complexes formed with Endo-S-treated IgG retain the ability to activate FcγRs in an IgG subclass-dependent manner. Human IgG1 and IgG3 antibodies deglycosylated by Endo-S are found to be able to activate FcγRs (Kao et al., 2015). Thus, the therapeutic efficacy of the Endo-S administration may not be predictable where FcγR activation via multivalent immune complexes is involved in disease pathogenesis. The remarkable specificity of Endo-S for native IgG has also been exploited for engineering of IgG glycoforms as described below.

## Influence of Fc glycan structure on pharmacokinetics of IgG antibodies

Clearance has a critical impact on the efficacy of therapeutic antibodies. IgG antibodies are protected from rapid degradation in lysosomes through the neonatal Fc receptor (FcRn) recycling mechanism, which explains the long half-life of IgG antibodies in the serum (Roopenian and Akilesh, 2007). FcRn interacts with IgG at the C_H_2/C_H_3 interface, independently of the Fc glycan. Other receptors that are known to bind and clear proteins with specific glycans include the asialoglycoprotein receptor that binds to terminal galactose residues of *N*-glycans (Ashwell and Harford, 1982) and the mannose receptor that recognizes terminal mannose or GlcNAc sugars (Lee et al., 2002). High mannose glycoforms are frequently found in recombinant IgG antibodies produced from tissue culture CHO and murine cells (Goetze et al., 2011; Mimura et al., 2009; Zhang et al., 2016). Shorter half-lives have been demonstrated for IgG antibodies bearing high mannose-type glycans compared with those with the complex-type glycans in mice (Kanda et al., 2007) and human FcRn-transgenic mice (Liu et al., 2011). When therapeutic IgG1 or IgG2 antibody was administered in human subjects, the relative abundance of IgG glycoforms with terminal galactose or GlcNAc remained constant during 34 days after injection while high mannose glycoforms were selectively cleared more rapidly at lower intravenous doses (Goetze et al., 2011). Thus, the presence of high mannose glycoforms may compromise the efficacy of antibody therapeutics through enhanced clearance and/or possible immunogenicity elicited by uptake of immune complexes via the mannose receptor on macrophages/dendritic cells and the activation of the mannan-binding lectin pathway (Arnold et al., 2006; Jefferis, 2017). The Fab is also glycosylated in approximately 20% of polyclonal human IgG, and the Fab glycans can be of highly galactosylated and sialylated complex-type (Holland et al., 2006; Mimura et al., 2007) or of high mannose-type, depending on the location of the glycosylation site in the V_H_ region (Gala and Morrison, 2004; Radcliffe et al., 2007; Wright et al., 1991). As Fab glycosylation can modulate the antibody binding (Wright et al., 1991) and physicochemical properties (Wu et al., 2010) and* in vivo* clearance as observed for highly glycosylated Fc-fusion proteins (Higel et al., 2016; Liu, 2015, 2017), the variable region glycosylation may also be exploited to improve the efficacy of antibody therapeutics.

## Biological activity of core fucose residue

The impact of fucose depletion from the IgG-Fc glycan on ADCC probably represents one of the most important discoveries in antibody glycobiology. The dramatic enhancement of ADCC is attributed to the improved affinity of nonfucosylated IgG for FcγRIIIa expressed on natural killer (NK) cells (Kanda et al., 2007; Okazaki et al., 2004; Shields et al., 2002; Shinkawa et al., 2003; Yamane-Ohnuki et al., 2004). In the past century, the biological relevance of core fucosylation received relatively little attention, in part, due to difficulty in the removal of core fucose from the IgG-Fc. Although the influence of the fucose residue on the stability of the Fc was examined by differential scanning calorimetry, fucosylated and nonfucosylated human IgG1-Fc proteins did not show any significant difference in the stability (Mimura et al., 2000; Mimura et al., 2001). However, the discovery of the importance of fucose depletion needed an appropriate binding partner, i.e., glycosylated (mammalian cell-expressed) FcγRIII. On the other hand, preparation of biologically active aglycosylated (*E*. *coli*-expressed) FcγRs was optimized in the late 1990s (Sondermann and Jacob, 1999), which led to the first crystallographic analyses of FcγRIIb (Sondermann et al., 1999) and the Fc–sFcγRIII complex (Sondermann et al., 2000). When the binding of fucosylated Fc to aglycosylated FcγRIII was analyzed by surface plasmon resonance (Maenaka et al., 2001), the Fc affinity to aglycosylated FcγRIII was slightly higher than that to glycosylated (CHO cell-expressed) FcγRIII observed by analytical ultracentrifugation (Ghirlando et al., 1995). Therefore, it was presumed at this time that *N*-glycosylation of FcγRIII negatively influences the Fc–FcγRIII interaction. Rather than core fucose, bisecting GlcNAc drew attention because recombinant IgG1 bearing bisected glycans by overexpression of β1,4-N-acetylglucosaminyltransferase III (GnT-III) exhibited improved ADCC (Umana et al., 1999), which later proved to be due to inhibition of α(1-6)-fucosyltransferase (FUT8) by the presence of bisecting GlcNAc.

Biological relevance of core fucose in the Fc glycan was demonstrated by two groups (Shields et al., 2002; Shinkawa et al., 2003). The Genentech group expressed glycosylated FcγRI, FcγRIIa, FcγRIIb and FcγRIIIa in mammalian cell lines (Shields et al., 2001) and humanized anti-HER2 and anti-IgE IgG1 antibodies with low fucose contents (ca. 10% and 21%, respectively) in Lec13 cells, a variant CHO cell line deficient in fucosylation (Shields et al., 2002). Lack of core fucose in the Fc glycan enhanced the binding of IgG to FcγRIIIa up to 50-fold, together with slightly improved binding to the Arg131 FcγRIIa polymorphic form and FcγRIIb whereas the absence of fucose did not affect the binding to human FcγRI, C1q, and neonatal FcR. The BioWa (or Kyowa Hakko Kirin) group produced an anti-CD20 antibody with low fucose contents (9%) in rat YB2/0 B-lymphoblast cells, and the antibody exhibited enhanced ADCC using human peripheral blood mononuclear cells (Shinkawa et al., 2003). The latter group has also found that increased bisecting GlcNAc contents in the nonfucosylated IgG glycans fractionated by PHA-E4 lectin affinity chromatography do not show additive effect on ADCC, which suggests that it is not bisecting GlcNAc but core fucose that markedly influences ADCC. The study also demonstrated the low expression level of *FUT8* mRNA in the YB2/0 B-cells, which led to the establishment of *FUT8* double gene-knockout CHO/DG44 cell line for production of completely nonfucosylated antibodies (Yamane-Ohnuki et al., 2004). It is known that human IgG1 binds more strongly to NK cells expressing homozygous FcγRIIIa-Val158 than to those expressing FcγRIIIa-Phe158 (Koene et al., 1997; Wu et al., 1997). IgG devoid of core fucose show improved binding to both FcγRIIIa-Val158 and FcγRIIIa-Phe158, with the affinity being increased up to 50-fold and 30-fold, respectively (Ferrara et al., 2006; Shields et al., 2002). Importantly, the glycan at Asn162 of FcγRIIIa is crucial for the high affinity of nonfucosylated IgG to FcγRIIIa whereas the glycan at Asn45 is required for proper folding but has a negative effect on the binding (Shibata-Koyama et al., 2009). X-ray crystallographic analysis of nonfucosylated Fc fragments produced in the *FUT8* ^−/−^ CHO/DG44 cells revealed a similar structure to the fucosylated counterpart (PDB ID codes: 2DTQ and 2DTS), with subtle difference in conformation around Tyr296 near the fucose residue. The stable-isotope-assisted NMR analyses also confirmed the similarity of the overall structures in solution (Matsumiya et al., 2007).

**Figure 3:**
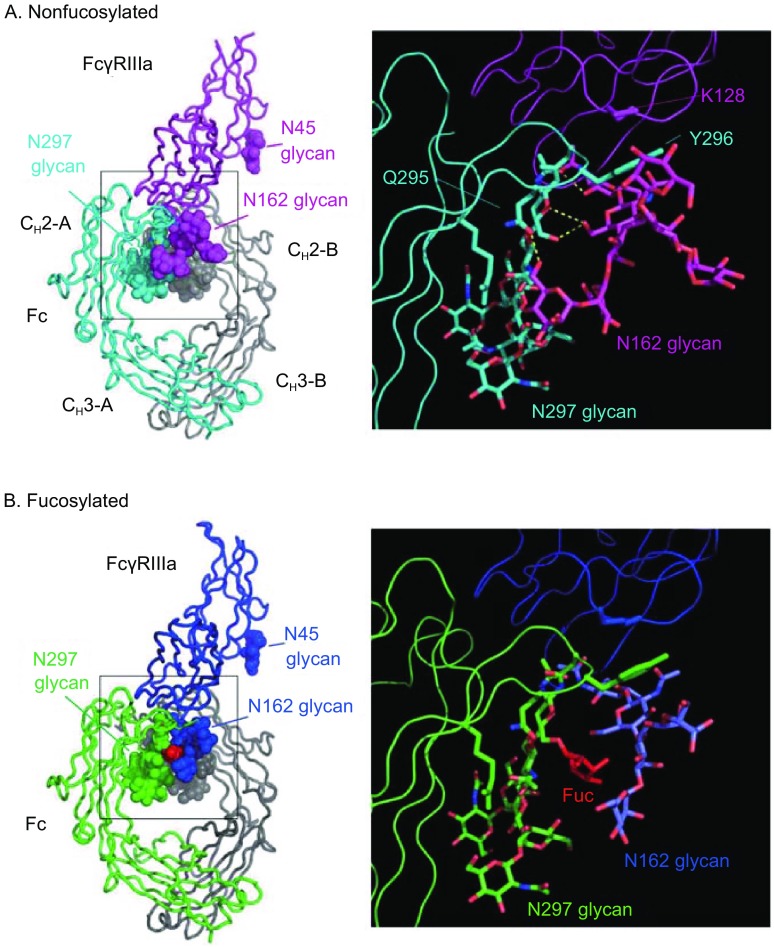
**Crystal structures of the complexes between FcγRIIIa and nonfucosylated Fc (A) or fucosylated Fc (B)**. (A) The nonfucosylated Fc chains A and B are shown in cyan and gray, respectively, and the FcγRIIIa in pink. (Right) The close-up view of the interaction interface between nonfucosylated Fc and glycosylated FcγRIIIa. (B) The fucosylated Fc chains are shown in green and gray, and the FcγRIIIa in blue. (Right) The close-up view of the interaction interface between fucosylated Fc and glycosylated FcγRIIIa. The oligosaccharides are shown in sphere (Left) and stick (Right) representation. Hydrogen bonds are presented as dashed lines. The molecular models were produced with PyMOL (The PyMOL Molecular Graphics System, Version 1.8.5.0, Schrodinger, LLC)

Crystal structures of the complex between nonfucosylated Fc and glycosylated FcγRIIIa have been solved by two independent groups (Ferrara et al., 2011; Mizushima et al., 2011). The crystal structure of the complex from Ferrara et al. was obtained with human nonfucosylated IgG1 produced from CHO-K1SV cells that overexpressed GnT-III to block the action of FUT8 and the sFcγRIIIa variant glycosylated at Asn45/Asn162 produced in HEK293-EBNA cells treated with the mannosidase I inhibitor, kifunensine (PDB ID code: 3SGK). The sFcγRIIIa bearing oligomannose glycans at the two sites binds nonfucosylated IgG1 with comparable affinity to the fully glycosylated FcγRIIIa. The crystal structure reveals unique interactions between the nonfucosylated glycan of the Fc and the high mannose-type glycan at Asn162 of sFcγRIIIa. The absence of core fucose allows hydrogen bonding between the chitobiose core of the glycan at Asn162 of sFcγRIIIa and the primary GlcNAc of the Fc glycan of the chain A (Fig. 3A). The terminal mannose residue on the α(1-3)-arm of the high mannose-type glycan of sFcγRIIIa forms a hydrogen bond to the Gln295 residue of the Fc. In addition, Tyr296 of the Fc makes contacts between the branching β-mannose and Lys128 residue of sFcγRIIIa (Fig. 3A). On the other hand, the crystal structure of the fucosylated Fc–glycosylated sFcγRIIIa complex (PDB ID code: 3SGJ) reveals that core fucose inhibits the ligand–receptor binding, due to steric hindrance (Fig. 3B). The other crystal structure of the nonfucosylated Fc–FcγRIII complex from Mizushima et al. was obtained with nonfucosylated IgG from the *FUT8* ^−/−^ CHO cells (Ms704) and sFcγRIIIa glycosylated at Asn45/Asn162 from CHO/DG44 cells (PDB ID code: 3AY4) (Mizushima et al., 2011). As revealed in the former crystal structure, the binding is mediated by the carbohydrate–carbohydrate and carbohydrate–protein interactions although this sFcγRIIIa bears biantennary fucosylated complex-type glycans, in contrast to high mannose-type glycans for the former. The glycan at Asn162 of sFcγRIIIa interacts with the nonfucosylated glycan and the Tyr296 and Arg301 residues of the Fc, thereby stabilizing the complex formation. These crystal structures demonstrate that the ADCC activity of nonfucosylated IgG is enhanced by the carbohydrate–carbohydrate interactions through van der Waals force, hydrogen bonding, and hydrophobic interactions. This is a novel mode of the ligand–receptor binding that provides an opportunity to explore optimal combinations of glycoforms of a ligand with those of a receptor to design glycosylated biological therapeutics.

Several nonfucosylated IgG antibodies have already entered the clinic. The phase I clinical trial of nonfucosylated humanized anti-CC chemokine receptor 4 (CCR4) IgG1 antibody KW-0761 (mogamulizumab, Fig. 1D) was initiated in patients with relapsed adult T cell leukemia or peripheral T-cell lymphoma in 2006 (Yamamoto et al., 2010). The phase II clinical trial demonstrated potent antitumor activity and tolerable toxicity profile by mogamulizumab monotherapy (Ishida et al., 2012), which led to approval by the regulatory authority in Japan in 2012. Several other nonfucosylated IgG antibodies are under clinical evaluation, including the ones against OX40 (KHK4083), IL-5R (benralizumab) (Wang et al., 2017), EGFR (imgatuzumab) (Delord et al., 2014), and CD20 (obinutuzumab). Fucose depletion of existing antitumor therapeutic IgG antibodies such as rituximab and trastuzumab has been shown to enhance the ADCC activities ex vivo (Iida et al., 2006; Mossner et al., 2010). The anti-CD20 humanized IgG1 antibody obinutuzumab produced in CHO-K1 cells engineered to overexpress GnT-III and Golgi β-mannosidase II exhibits low fucose contents in the Fc glycans and superior antitumor activities to rituximab (Sehn et al., 2012; Sehn et al., 2015). Obinutuzumab was approved in the United States in 2013 for treatment of follicular lymphoma. Thus, nonfucosylated IgG antibodies will be further developed as next-generation therapeutic antibodies with potent ADCC at reduced doses.

## Biological activity of the terminal sialic acid residues

**Figure 4:**
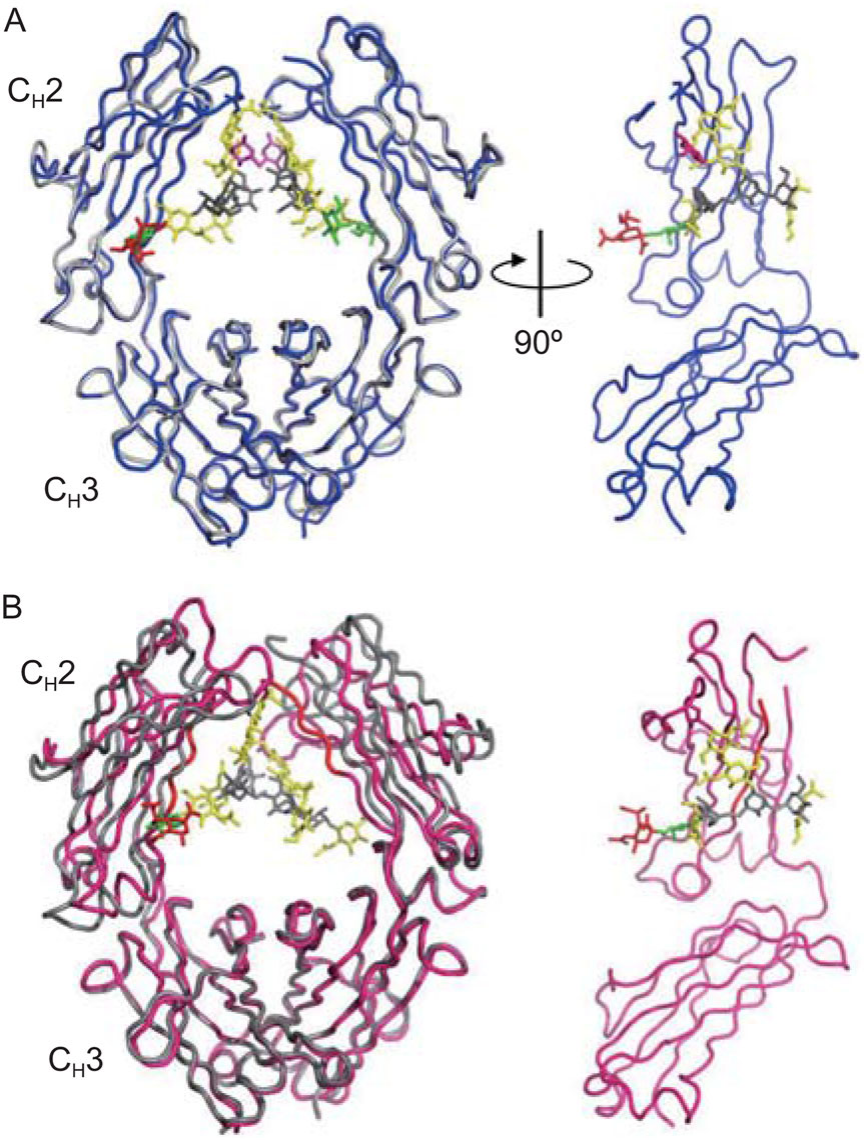
**Comparison of sialylated and native Fc structures**. (A) Superposition of enzymatically sialylated Fc (blue) (PDB ID code: 4BYH) and native Fc (gray) (PDB ID code: 1H3Y). (B) Superposition of chemoenzymatically synthesized disialylated Fc (magenta) (PDB ID code: 4Q6Y) and native Fc (gray) (PDB ID code: 3AVE). The monosaccharides fucose, GlcNAc, mannose, galactose, and sialic acid are shown in magenta, yellow, gray, green and red, respectively. The molecular models were produced with PyMOL (The PyMOL Molecular Graphics System, Version 1.8.5.0, Schrodinger, LLC)

Influence of sialylation on the structure of the Fc has been analyzed by NMR and X-ray crystallographic analysis (Ahmed et al., 2014; Barb et al., 2009; Barb et al., 2012; Crispin et al., 2013). By NMR spectroscopy the relaxation rates of the galactose resonances for the Fc monosialylated on the α(1-3)-arm and the disialylated Fc were found to be largely similar to those for the G2F Fc glycoform, which suggests that Fc sialylation has a minor effect on the motional behavior of the *N*-glycan. Although the sialic acid residues are highly dynamic and free of strong interaction with the protein moiety of the Fc, the sialylated glycan–C_H_2 polypeptide interactions are largely mediated by the carbohydrate residues up to galactose (Barb et al., 2012). The crystal structure of sialylated Fc (PDB ID code: 4BYH) provides consistent findings with the solution-state NMR measurements (Fig. 4A). The terminal sialic acid on the α(1-6)-arm (Fig. 4A, shown in red) projects away from the protein surface in a solvent-exposed manner, and the monosaccharides on the α(1-3)-arm are visible up to GlcNAc for the Fc chains (Crispin et al., 2013). The lack of electron density for terminal sialic acid residues on the α(1-3)-arm is consistent with the dynamics of the terminal sialic acid observed by the NMR study. This crystal structure of the enzymatically sialylated Fc does not show gross conformational change as compared with that of the native Fc (PDB ID code: 1H3Y, Fig. 4A) whereas those of the disialylated Fc fragments prepared by chemoenzymatic glycoengineering show both open and closed C_H_2 domain conformations in the crystal (Fig. 4B, PDB ID code: 4Q6Y) (Ahmed et al., 2014). The distances between the C^α^ atoms at the Pro238 residues of the C_H_2 domains for the open and closed conformers of the disialylated Fc and a representative native Fc (PDB ID code: 3AVE) are 13 Å, 20.2 Å and 19.3 Å, respectively. Although it is unclear whether the conformational heterogeneity of the disialylated Fc results from sialylation or crystal packing contacts, increased conformational flexibility of the sialylated Fc may be associated with anti-inflammatory properties of this glycoform as described below (Ahmed et al., 2014).

Sialylated glycans of the Fc have recently drawn increased attention as an active component of IVIG that exerts anti-inflammatory properties. IVIG has been used to treat not only immunodeficiency (hypogammaglobulinemia) but also various autoimmune diseases including idiopathic thrombocytopenic purpura and Kawasaki disease. It has been shown that the anti-inflammatory effects of IVIG reside in the Fc region of IVIG and that infusion of Fc fragments ameliorates the conditions of children with acute immune thrombocytopenic purpura (Debre et al., 1993). It has been proposed that IVIG binds to an inhibitory FcγRIIb that transmits an inhibitory signal in the cytoplasm to suppress inflammation. The protective effect of IVIG was associated with the induced expression of an inhibitory Fc receptor FcγRIIb (Bruhns et al., 2003) although a correlation between the sialylation level and the anti-inflammatory activity of IgG was not provided.

Multiple effects of Fc sialylation on antibody effector functions and the immune system have been reported including reduction of ADCC (Kaneko et al., 2006; Scallon et al., 2007) and CDC (Quast et al., 2015) and induction of T_H_2 cytokine IL-33 and upregulation of FcγRIIb (Anthony et al., 2011). Sialylated forms of IgG enriched with *Sambucus nigra* agglutinin (SNA) show reduced affinity to FcγRIII, thereby reducing the ability of the IgG autoantibodies to trigger* in vivo* cytotoxicity (Kaneko et al., 2006). In addition to this inhibitory effect, SNA-enriched IVIG-Fc is shown to exert anti-inflammatory activity in the murine K/BxN serum transfer arthritis model equivalent to unfractionated IVIG-Fc at a 10-fold lower dose. The anti-inflammatory activity of SNA-enriched IVIG has been recapitulated with highly sialylated, recombinant human IgG1-Fc (Anthony et al., 2008a). The receptor required for the anti-inflammatory effect of the sialylated IgG has been identified as the C-type lectin, SIGN-R1, expressed on murine splenic macrophage (Anthony et al., 2008b), and its human orthologue DC-SIGN has been shown to act as a receptor for sialylated IgG in human DC-SIGN transgenic mice (Anthony et al., 2011). The proposed mechanism by which sialylated IgG exerts anti-inflammatory effects is T_H_2 cytokine IL-33 expression in SIGN-R1^+^ or DC-SIGN^+^ macrophages/dendritic cells through interaction with sialylated IgG. IL-33 then suppresses inflammation by induction of IL-4 from basophils which leads to upregulation of inhibitory receptor FcγRIIb on effector macrophages. However, the anti-inflammatory activity of sialylated IgG has not been reproduced in some mouse models of autoimmune diseases. No differences were observed between SNA-enriched IVIG and neuraminidase-treated IVIG in the efficacy to ameliorate ITP (Guhr et al., 2011; Leontyev et al., 2012b), K/BxN serum transfer arthritis (Campbell et al., 2014), and experimental autoimmune encephalomyelitis (Othy et al., 2014). In the K/BxN serum arthritis model, depletion of basophils did not influence the anti-inflammatory effect of IVIG. The requirement of FcγRIIb for anti-inflammatory effects of IVIG was not demonstrated by using FcγRIIb-knockout mice (Bazin et al., 2006; Leontyev et al., 2012a). Furthermore, neither sialylated nor native Fc was shown to bind to recombinant DC-SIGN although the binding of serum IgG and its deglycosylated, desialylated, and sialylated glycoforms to DC-SIGN was comparable, indicating that the DC-SIGN binding to IgG could be attributed to cross-reactive, polyclonal Fab specificities (Yu et al., 2013). It has been shown that F(ab′)_2_ fragments of IVIG could directly interact with DC-SIGN on dendritic cells, which ultimately leads to expansion of Treg cell populations (Trinath et al., 2013). It seems that the discrepancies of the anti-inflammatory effects of sialylated IgG among these studies have arisen due to different IVIG-Fc preparations at differing sialylation levels in the presence or absence of F(ab′)_2_, different glycan analysis methods and different genetic backgrounds of mice. Another key question exists around the anti-inflammatory properties of IVIG regarding the identification of the human counterpart of the DC-SIGN^+^ macrophage from the DC-SIGN-transgenic mouse. Thus, the impact of sialylation of IgG-Fc on immunosuppression in autoimmune diseases remains unsolved, and further studies are needed to elucidate the mechanism of action of IVIG.

## Chemoenzymatic glycoengineering

**Figure 5:**
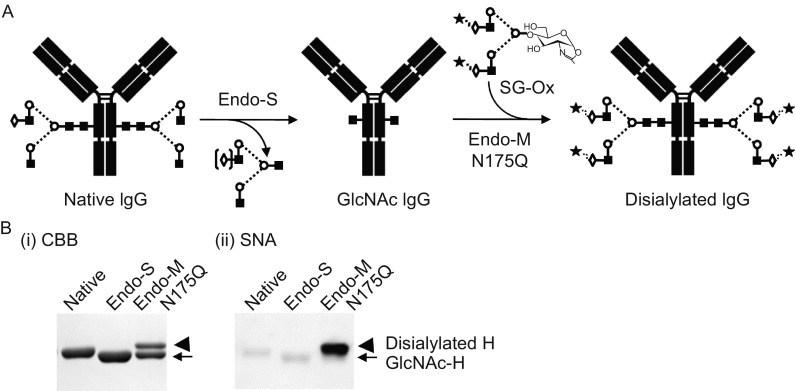
**Glycosylation remodeling of IgG using Endo-S and Endo-M-N175Q**. (A) Schematic representation of the chemoenzymatic glycoengineering method. SG-Ox, sialoglycan-oxazoline. (B) SDS-PAGE of the transglycosylation product of IgG. Mogamulizumab (1 mg) was deglycosylated with Endo-S (2,000 U, New England Biolabs) in 50 mmol/L acetate containing 5 mmol/L CaCl_2_ (pH 5.5) and purified on a protein G column. The deglycosylated IgG (0.5 mg) was incubated with Endo-M-N175Q (100 mU, Tokyo Chemical Industry, Japan) and 1 mg of SG-Ox (Fushimi Pharmaceutical, Japan) in 50 μL of 50 mmol/L sodium phosphate (pH 6.5) at 30°C for 2 h. CBB, Coomassie brilliant blue; SNA, *Sambucus nigra* agglutinin

Separation of various glycoforms to investigate the biological relevance of glycosylation is a real challenge with glycoproteins. Although cell engineering through overexpression or disruption of relevant enzyme genes have been employed to produce specific glycoforms of IgG (Ha et al., 2011; Li et al., 2006; Raymond et al., 2015; Umana et al., 1999; Yamane-Ohnuki et al., 2004), it is still challenging to optimize the production of desired glycoforms of IgG. Recently, transglycosylation reactions have been applied to synthesis of new glycoconjugates that consist of deglycosylation by an endo-β-N-acetylglucosaminidase (ENGase) to leave the innermost GlcNAc with or without core fucose at the *N*-glycosylation site(s) and subsequent reglycosylation by an ENGase-based glycosynthase to transfer a predefined *N*-glycan substrate to the innermost GlcNAc (Giddens and Wang, 2015; Huang et al., 2012; Umekawa et al., 2010) (Fig. 5). This technique utilizes highly active glycan oxazolines, the mimics of the transition state, as donor substrates (Kobayashi et al., 1996), and transglycosylation with the synthetic glycan oxazoline proceeds in both a stereo- and regiospecific manner (Li et al., 2005). This chemoenzymatic glycoengineering is recognized as one of the most promising approaches to synthesize homogeneous glycoforms of a given glycoprotein including IgG and has been applied to the synthesis of fully sialylated IgG glycoforms which would otherwise be quite difficult (Ahmed et al., 2014; Kurogochi et al., 2015; Lin et al., 2015).

Several ENGases possess transglycosylation activity, including Endo-A from *Arthrobacter protophormiae* (Takegawa et al., 1995; Takegawa et al., 1997), Endo-M from *Mucor hiemalis* (Fujita et al., 2004; Yamamoto et al., 1994), Endo-D from *Streptococcus pneumoniae* (Fan et al., 2012; Muramatsu et al., 2001), and Endo-CE from *Caenorhabditis elegans* (Kato et al., 2002) in the glycoside hydrolase (GH)-85 family and Endo-S from *Streptococcus pyogenes* (Huang et al., 2012) and Endo-S2 from *Streptococcus pyogenes* of serotype M49 (Li et al., 2016) in the GH18 family. Various glycosynthase mutants of ENGases have been generated to abolish the hydrolytic activity on the transglycosylation products and improve the transglycosylation efficiency, including Endo-A-N171A, Endo-M-N175Q (Fig. 5B), Endo-S-D233Q, and Endo-S2-D184M. Different ENGases have distinct substrate specificity and limitations. Endo-M acts on both the complex-type and high mannose-type oligosaccharides whereas Endo-A and Endo-S are limited to action on the high mannose-type and the complex-type, respectively. In addition, Endo-S and Endo-S2 in GH18 act on both nonfucosylated and fucosylated glycans whereas ENGases in GH85 are generally inactive on fucosylated glycans except Endo-D. The substrate specificity of an ENGase for transglycosylation is not always the same as that of the wildtype ENGase for hydrolysis, e.g., Endo-D prefers fucosylated glycans for hydrolysis whereas its N322Q mutant favors the nonfucosylated GlcNAc as the acceptor (Fan et al., 2012). The ENGase-catalyzed transglycosylation has further been improved through the efficient synthesis of sugar oxazolines in H_2_O with 2-chloro-1,3-dimethylimidazolinium chloride (Noguchi et al., 2009) or 2-chloro-1,3-dimethyl-1H-benzimidazol-3-ium chloride (CDMBI) as a dehydrative condensing agent (Noguchi et al., 2012).

Chemoenzymatic glycosylation of IgG antibodies has been reported with Endo-A, yeast-produced IgG-Fc bearing high mannose-type glycans as the acceptor and Man_3_GlcNAc-oxazolines as the donor substrate (Wei et al., 2008). Complete transglycosylation of the IgG-Fc with Endo-A required a large excess quantity of the donor substrate. Two mutants of Endo-D (N322A and N322Q) can also attach a Man_3_GlcNAc tetrasaccharide to a fucosylated GlcNAc-containing Fc (Fan et al., 2012) whereas none of Endo-D, Endo-A and their mutants can transfer intact complex-type *N*-glycan to either fucosylated or non-fucosylated GlcNAc-containing Fc. The Endo-M-N175Q mutant has recently been shown to act on proteins with a broad range of molecular weight including IgG (Fig. 5) despite a preference of low molecular weight proteins as acceptors. Endo-S mutants (Endo-S D233A and D233Q) are the first ENGase-based glycosynthases applicable for remodeling of IgG glycoforms with fucosylated and nonfucosylated full-length complex-type glycans using rituximab (Huang et al., 2012). The D184M and D184Q mutants of Endo-S2 from *Streptococcus pyogenes* NZ131 (serotype M49) have been reported to have more potent transglycosylation activity and more relaxed substrate specificity than the Endo-S-D233Q mutants (Li et al., 2016). Among the high mannose-, hybrid-, and complex-type *N*-glycan substrates, Endo-S2 prefers the complex-type over the other two types. The lower concentrations of sugar oxazolines and the shorter incubation times would be beneficial to reduce the risk of unwanted side reactions to the transglycosylation products.

Industrial scale production of homogeneous antibody glycoforms by chemoenzymatic glycoengineering would require large-scale production of homogeneous oligosaccharide substrates, simplification of synthesis for sugar oxazolines, and enhancement of the transglycosylation efficiency of glycosynthases. The production of complex-type oligosaccharides has recently been developed using egg yolk (Sun et al., 2014). The one-step synthesis of sugar oxazolines from unprotected sugars has been discovered by the use of CDMBI (Noguchi et al., 2012). The transglycosylation efficiency of the ENGase-based glycosynthases has been improved by systematic mutagenesis at the critical residues of various ENGases (Li et al., 2016). The development of this glycoengineering technology opens a new avenue to glycoform remodeling for therapeutic purposes.

## Conclusion

IgG-Fc glycoengineering contributes to the development of next-generation therapeutic IgG antibodies with enhanced or silenced Fc effector functions. With the success of nonfucosylated IgG antibodies in the clinic, glycoengineered IgG antibodies have proven to be efficacious and devoid of immunogenicity* in vivo* as long as the Fc bears naturally occurring human-type glycans, in contrast to mutant forms of antibodies. Therefore, a range of glycan structures from monosaccharide GlcNAc to fully sialylated biantennary complex-type are to be explored for the design of homogeneous IgG glycoforms as therapeutic antibodies. Chemoenzymatic glycoengineering is a robust approach for remodeling of IgG-Fc glycoforms. It should be noted that gain- or loss-of-function may occur in a subclass-dependent manner as human IgG consists of four subclasses with differing abilities to activate different FcγRs and complement (Kao et al., 2015; Niwa et al., 2005). As the structural basis for the enhanced ADCC activity of nonfucosylated IgG antibodies has been elucidated, the carbohydrate–carbohydrate interactions between IgG-Fc and FcγR can also be a key issue for the design of novel glycoengineered IgG antibodies. On the other hand, *E*. *coli*-produced aglycosylated IgG antibodies with compromised effector functions can be exploited as neutralizing, agonist and antagonist antibodies for a wide range of diseases including cancers and autoimmune diseases. Bypassing glycosylation contributes to shorter bioprocess development and running times, without concerns about glycosylation heterogeneity as CQAs, and is expected to substantially reduce production costs. The high costs of therapeutic antibodies have imposed financial pressures on national and private health care bodies. Blocking/neutralizing antibodies anti-PD-1 IgG nivolumab (Fig. 1B), anti-VEGF IgG bevacizumab (Fig. 1C), and anti-TNFα IgG infliximab are among largest selling pharmaceuticals that could maintain their efficacies in an aglycosylated format as demonstrated by the licensed therapeutic Fab fragments certolizumab and ranibizumab specific for TNFα and VEGF-A, respectively. Thus, glycoengineering provides strategies to optimize the safety, functionality, and efficacy of therapeutic IgG antibodies as more affordable treatment options in the next decade.
